# Lipolymphedema Associated with Idiopathic Cyclic Edema: A Therapeutic Approach

**DOI:** 10.1155/2017/5470909

**Published:** 2017-08-23

**Authors:** Jose Maria Pereira de Godoy, Henrique Jose Pereira de Godoy, Aline Aparecida de Sene Souza, Ricardo Budtinger Filho, Maria de Fatima Guerreiro Godoy

**Affiliations:** ^1^Cardiology and Cardiovascular Surgery Department, The Medicine School in São José do Rio Preto (FAMERP), Sao Jose do Rio Preto, SP, Brazil; ^2^Clínica Godoy, São José do Rio Preto, SP, Brazil; ^3^Universidade Federal do Mato Grosso, Cuiabá, MT, Brazil; ^4^Research Group, Clínica Godoy, São José do Rio Preto, SP, Brazil; ^5^The Medicine School in São José do Rio Preto (FAMERP), Sao Jose do Rio Preto, SP, Brazil

## Abstract

Idiopathic cyclic edema is a type of generalized edema that mainly affects women. Diagnosis is made by the patient's clinical history and an evaluation of the accumulation of weight during the day. The objective of this study is to report the clinical control of lymphedema associated with idiopathic cyclic edema using calcium dobesilate. A 55-year-old female patient reported generalized edema for years in that she woke up in the morning with her legs swollen and the edema worsened during the day. The physical examination revealed generalized edema. After four days of treatment with calcium dobesilate, the patient returned to the Clínica Godoy, Brazil, with less edema and reductions in body weight and the amount of extracellular and intracellular fluid. With further treatment, there was a total reduction of the edema. It is concluded that calcium dobesilate helps to control lymphedema secondary to idiopathic cyclic edema.

## 1. Introduction

Lymphedema is a type of edema caused by a failure in the formation and/or drainage of lymph that results in an accumulation of macromolecules and fluids in the interstitial space [1]. Lymphedema is classified as primary and secondary etiopathogenesis. In primary lymphedema, alterations exist at birth and in secondary disease; the patient suffers lesions to the lymphatics during life due to inflammatory or infectious processes, neoplasia, or traumas [1, 2]. Furthermore, the clinical classification is divided into four stages, where stage 0 is subclinical and in stage I there is no edema in the morning, however, during the day edema develops with a lessening of the swelling on resting with the legs elevated. Stage II is characterized by edema in the morning, with no improvement with rest and worsening during the day. Finally, stage III is the most advanced phase of lymphedema, popularly known as elephantiasis, which is identified by deformities of the affected region [1, 3].

Lipedema is an inherited condition that is characterized by bilateral and symmetrical enlargement of the lower limbs. It can be accompanied by symptoms such as hyperalgesia and cutaneous hypothermia. When lipedema progresses to lymphedema, it is called lipolymphedema. On the other hand, idiopathic cyclic edema is a clinical condition related to vessel hypermeability, associated with increased interstitial fluid and characterized by generalized edema [3]. Idiopathic cyclic edema is a factor that can aggravate edema in patients with lipedema and lymphedema [4, 5].

These changes can cause complications for patients, for both the physiology and personal relationships in the social sphere. Thus, early diagnosis and treatment are essential for a good prognosis and the patient's well-being. The objective of this study is to report the clinical control of a case of lipolymphedema associated with idiopathic cyclic edema using calcium dobesilate.

## 2. Case Report

Herein we report a case of a 55-year-old female patient who sought medical attention complaining of generalized “swelling,” mainly of the hands and feet. The edema had begun at the ankles 35 years previously when she was pregnant. According to the patient, the edema worsened at the end of the day. Over the years, the edema spread to the hands and wrists and currently affects the entire body causing abdominal discomfort at the end of the day. Over the years, the patient sought several medical specialists to reduce the symptoms of edema. However, no effective results were obtained in spite of the therapeutic approaches, mainly with diuretics. She was referred to the Clínica Godoy, Brazil, where the medical history was taken and a physical examination was performed. Generalized edema was identified, especially in the lower limbs, which was present in the early morning and worsened during the day especially on hot days. Another important fact reported by the patient was the weight gain of 1 to 2 kg by the end of the day. A bioimpedance test was performed using the* In Body S10* apparatus, which detected a large amount of extracellular fluids in the entire body and intracellular fluids at the upper limit of normality. By bioimpedance, the volume of fluids in the lower extremities and trunk was above the upper limits, as was the ratio between extracellular fluids and total fluids which characterizes edema (Table 1). The upper limbs had a higher than normal volume of fluids, but without characterizing edema. After analysis, the diagnosis was idiopathic cyclic edema, clinical stage II lower limb lymphedema, lipedema stage I and excessive weight. Calcium dobesilate b.i.d. was prescribed and after four days another bioimpedance was performed that showed a significant reduction of the fluids of the upper limbs and trunk that was within the range of normality for the whole body. This study was approved by the Research Ethics Committee of the Medical School in São José do Rio Preto (#048856/2016, CAAE 56497016.6.0000.5415).

## 3. Discussion

The present study describes the evolution of edema to clinical stage II lymphedema over time in a patient with lipedema and idiopathic cyclic edema. The clinical stage II lymphedema in this case was reversed using calcium dobesilate. No study reporting these findings was found in the literature.

Patients with lipedema may present two distinct pathophysiological conditions, lymphostasis and necrosis of fatty tissue. Idiopathic cyclic edema leads to altered capillary permeability with an overload of the lymphatic system, which is the functional reserve of the venous system. Long-term evolution may lead to a hyperdynamic-type lymphedema as occurring in this patient, resulting in lipolymphedema. Therefore, there is an association of lymphostasis aggravated by overload related to idiopathic cyclic edema.

The diagnosis of idiopathic cyclic edema is basically clinical; there is an increase of more than 800 grams at the end of the day [4]. This edema becomes generalized under the influence of gravitational pressure. In the morning, edema of the face and difficulty to remove rings can be reported with these signs normalizing during the course of the day; however, in the evening the patient has edema of the legs and abdominal discomfort.

Control of cyclic edema is achieved using medications, but these patients cannot ingest excessive quantities of fluids; they should drink only when they are thirsty [5]. This is important or else the medication will not control the edema. The therapeutic response is almost immediate and the reduction of edema is observed the day after. We usually advise patients to weigh themselves in the morning and afternoon under similar conditions, that is, with an empty stomach and bladder. In the control of the medication a reduction of the weight variation to less than 300 grams must be observed, as occurring in this patient.

This case is important in the treatment of lymphedema in women because from 5 to 10% of women with lymphedema who seek the clinic have idiopathic cyclic edema. The control of cyclic edema is fundamental; otherwise, control of lymphedema is more difficult because it will be impossible to control the excessive fluid production that leads to an overload of the lymphatic system.

In this patient, control of cyclic edema led to control of the lymphedema. The objective in this case was to evaluate the efficacy of calcium dobesilate, but the medication could have been associated with lymph drainage and compression therapy to accelerate the reduction of the edema.

## 4. Conclusion

The control of idiopathic cyclic edema is essential in the treatment of hyperdynamic lymphedema due to fluid overload. Calcium dobesilate can help in the control of idiopathic cyclic edema.

## Figures and Tables

**Table 1:** Table 1 shows the total intracellular and extracellular fluid volume of the limbs and trunk before and after treatment and the ratio of extracellular water to total water.

Evaluation site	12-5-2016	28-6-2016	Normal values
Total intracellular fluid, patient (L)	22.0	20.8	18.5–22.5
Total intracellular fluid, patient (L)	14.7	13.2	11.3–13.9
Right arm fluid (L)	1.95	1.85	1.50–1.84
Left arm fluid (L)	1.94	1.79	1.50–1.84
Right leg fluid (L)	6.51	5.75	4.77–5.83
Left leg fluid (L)	6.44	5.58	4.77–5.83
Extracellular fluid/total fluid ratio	0.399	0.387	0.36–0.39
Extracellular fluid/total right arm fluid ratio	0.387	0.385	0.36–0.39
Extracellular fluid/total left arm fluid ratio	0.389	0.385	0.36–0.39
Extracellular fluid/total right leg fluid ratio	0.404	0.389	0.36–0.39
Extracellular fluid/total left leg fluid ratio	0.405	0.390	0.36–0.39
Extracellular fluid/total trunk fluid ratio	0.397	0.386	0.36–0.39

*Note*. Extracellular fluid/total fluid ratio defining lymphedema.
